# A protocol to assess cell cycle and apoptosis in human and mouse pluripotent cells

**DOI:** 10.1186/1478-811X-9-8

**Published:** 2011-04-11

**Authors:** Michael J Edel, Cristina Menchon, Jose Miguel Andres Vaquero, Juan Carlos Izpisua Belmonte

**Affiliations:** 1 Center of Regenerative Medicine in Barcelona, Dr. Aiguader 88, 08003 Barcelona, Spain; 2 Current Address: Vall d' Hebron Research Institute, Xcelia and Banc de Sang i Teixits, Passeig Vall d'Hebron, 119 - 129, 08035 Barcelona, Spain; 3 Current Address: Affiliation with Victor Chang Cardiac Research Institute, Lowy Packer Building 405 Liverpool St, Darlinghurst New South Wales 2010, Australia; 4 Gene Expression Laboratory, Salk Institute for Biological Studies, 10010 North Torrey Pines Rd., La Jolla, California 92037, USA

## Abstract

Embryonic stem cells (ESC) and induced pluripotent stem cells (iPSCs) present a great opportunity to treat and model human disease as a cell replacement therapy. There is a growing pressure to understand better the signal transduction pathways regulating pluripotency and self-renewal of these special cells in order to deliver a safe and reliable cell based therapy in the near future. Many signal transduction pathways converge on two major cell functions associated with self-renewal and pluripotency: control of the cell cycle and apoptosis, although a standard method is lacking across the field. Here we present a detailed protocol to assess the cell cycle and apoptosis of ESC and iPSCs as a single reference point offering an easy to use standard approach across the field.

## Introduction

Human embryonic stem cells (hESC) have been derived from the inner cell mass of blastocytes and can be maintained *in vitro *indefinitely with the addition of fibroblastic growth factor 2 (FGF2) [1]. It has been shown that a combination of three or four pluripotency transcription factors; Oct4, Sox2 and KLf4, with or without Myc can reprogram somatic cells to generate induced pluripotent stem cells (iPSC) [2,3]. Recently, it has been shown that loss of p53 function can enhance the efficiency of reprogramming, suggesting that the cell cycle is a rate limiting step in the reprogramming process [4-10]. Given the important role of the cell cycle and apoptosis in pluripotent cells, a detailed protocol that standardises the technique of measuring cell cycle and apoptosis in pluripotent cells across the field is called for.

The cell cycle of mouse ESCs and to a lesser extent hESCs has been well described [11-13]. The cell cycle regulatory machinery of ESC including the Cyclin families A, E, D, and B and their kinases CDK2, 4 and 6, are not regulated in a cyclic fashion, with the exception of cyclin B [11,12]. Of the core machinery, cyclin A/E/CDK2 are regarded as constitutively on in mouse ESC driving an almost non-existent G_1 _phase into S phase, in which 60-70% of ESC are present. Consequently, the rate of ESC proliferation is much faster with an average cycle lasting just twelve hours compared to somatic cells [13,14]. A recent study functionally demonstrated that Cyclin A regulates pluripotency but is redundant in fibroblasts suggesting it is a pluripotent associated cell cycle kinase [15]. Many of the peripheral genes that control cell cycle such as p16^INK4a ^are thought to be inactive, resulting in a different regulation of cell cycle in mouse ESC [16]. It is evident that there are many differences between somatic and pluripotent cells; however, little is known about the role of the cell cycle in maintaining ESC or iPSC in culture. Much less is known about control of apoptosis in ESC or iPSC while under *in vitro *conditions.

Here we describe a protocol how to measure cell cycle and apoptosis in pluripotent stem cells (hESC and iPS cells) that could be used, for example, following infection with a lentivirus to manipulate cell cycle function of ESC or iPSC *in vitro*. Detailed protocols how to make lentivirus have been published previously [17-19]. We describe a standard method to measure cell cycle profile by FACs in hESC and iPS cells using EdU substrate; and apoptosis using DilC mitochondrial membrane method in the same sample concurrently. The use of DNA dyes and EdU staining (an advance on BRDU staining) has been discussed extensively by Cappella et al and Hamelik et al [20,21]. In addition we describe a list of working antibodies for cell cycle and apoptosis proteins by western blot methodology. The protocol described here is applicable to any human or animal pluripotent stem cell and enables "getting the most" from your cell cycle and apoptosis analyses, offering an easy to reference, comprehensive standardised protocol across the field of pluripotent stem cells.

## Materials

For detailed methods on how to make iPSCs as well as how to infect such cells using concentrated lentiviruses and supernatant retroviruses including reagents, equipment and set up please refer to recently published work [17-19].

## Reagents

• Human and mouse embryonic stem cells (ESC)

CAUTION: Check ethic issues with your specific institute for use of hESC.

• Human or mouse induced pluripotent stem cells (iPSC)

• Mitotically inactivated mouse embryonic fibroblasts (irMEFs)

• Mitotically inactivated human foreskin fibroblasts (irHFFs) (ATCC, cat. no. CRL-2429)

• 0.1% (wt/vol) Gelatin solution (Millipore, cat. no. ES-006-B)

• Matrigel (Mg, BD Biosciences, cat. no. 356234)

• Dulbecco's PBS, without Ca2+ or Mg2+ (PAA laboratories GmbH, cat. no. H15-002)

• DMEM (Invitrogen, cat. no. 21969-035)

• KO-DMEM (Knockout DMEM, Invitrogen, cat. no. 10829-018)

• Fetal calf serum (FCS, Perbio, Hyclone, cat. no. CH30160.03)

• Heat-inactivated fetal bovine serum (FBS, Invitrogen, cat. no. 10270-106)

• Knockout Serum Replacement (KO-SR, Invitrogen, cat. no. 10828-028)

• Trypan Blue stain (Invitrogen, cat. no. 15250-061)

• 20% (wt/vol) Human serum albumin (HSA, Instituto Grifols SA, cat. no. 670612)

• GlutaMAX (Invitrogen, cat. no. 35050-038)

• Minimum essential medium Eagle's nonessential amino acids, 100 × (Lonza, cat. no. BE13-114E)

• Nucleosides, 100 × (Millipore, cat. no. ES-008-D)

• Penicillin/streptomycin (Invitrogen, cat. no. 15140-163)

• Lipofectamine 2000 (Invitrogen)

• 50 mM 2-mercaptoethanol (Invitrogen, cat. no. 31350-010)

• Basic fibroblast growth factor (Peprotech cat. no. 100-18B) (see REAGENT SETUP)

0.25% (wt/vol) Trypsin/EDTA (Invitrogen, cat. no. 25200-056)

0.05% (wt/vol) Trypsin/EDTA (Invitrogen, cat. no. 25300-054)

• Polybrene (10 mg ml-1, Millipore, cat. no. TR-1003-G)

• FuGENE 6 transfection reagent (Roche Applied Science, cat. no. 11 988 387 001)

• DMSO (Sigma, cat. no. D4540)

• MitoProbe DilC assay kit for flow cytometry (M34151)

• Invitrogen Click iT Edu cell proliferation assay kit for flow cytometry (A10202).

## Equipment

• DakoCytomation MoFlo High-performance cell sorter equipped with 3 lasers, an Innova 90C UV laser from Coherent, a red diode 635nm and a Lytcyt200s 488 nm solid-state laser. The PMT detectors used are Hamamatsu -15.

• Leica SP5 AOBS confocal microscope

• Water Bath set to 37°C.

• Class-II cabinet with aspirator for tissue culture (Telstar Bio-II-A)

• Class-II cabinet with space for stereomicroscope (Telstar Bio-II-A/G)

• Aspirator tube assembly (Sigma, cat. no. A5177)

• Stereomicroscope (Olympus SZX12, Olympus)

• Low-end color video camera (JVC TKC1481BEG)

• Low-end 8-inch LCD display (BOMAN TV304)

• Inverted tissue culture microscope with phase contrast and epifluorescence, with ×5, ×10, ×20 and ×40 objectives (Leica DMIL, Leica)

• Thermostatized tissue culture centrifuge and swinging rotor with adapters for 15-ml and 50-ml tubes and microplates (Beckman Coulter Allegra X-12R centrifuge with SX4750A rotor, Beckman Coulter)

• High-speed centrifuge and swinging rotor (Beckman Coulter Avanti J-30I centrifuge with JS-24.38 rotor, Beckman Coulter)

• Cell culture incubator set at 37°C, 5% CO2 (REVCO, cat. no. RCO3000D-9-VBC)

• Cell culture incubator set at 32°C, 5% CO2 (REVCO, cat. no. RCO3000D-9-VBC)

• Cell culture incubator set at 37°C, 5% CO2 and 5% O2 (REVCO, cat. no. RTG5000D-9-VBC)

• Microcentrifuge (Eppendorf 5424, Eppendorf)

• Tissue culture dish, 100 and 150 mm

• Tissue culture flasks, T75 and T150

• Tissue culture plates, six-well

• Conical tubes, 15 and 50 ml

• Screw-cap microcentrifuge tubes (Sarstedt, cat. no. 72.692.005)

• Polyallomer centrifuge tubes, 13 × 51 mm (Beckman, cat. no. 326819)

• Cover slips

• Stripper micropipette (Mid Atlantic, cat. no. MXL3-STR)

• Stripper tips, 150 uM (REF MXL3-150) ORIGIO mid-atlantic devices.

• Cryo 1°C Freezing Container, 'Mr. Frosty' (Nalgene, cat. no. 5100-0001)

• Slide flask (Nunc, cat. no. 170920)

• Bottle-top filter system 0.22 μm, 500 ml (Millipore, cat. no. SCGPU05RE)

• Filter, Mille-HV PVDF (polyvinylidene fluoride) (0.45 μm, Millipore, cat. no. SLHV033RS)

• Invitrogen western blot and transfer equipment.

## REAGENT SETUP

### Basic fibroblast growth factor (bFGF)

After spinning briefly, resuspend the contents of the vial with 10 ml of 0.2% (wt/vol) HSA in PBS. The final concentration of bFGF is 100 μg/ml. Prepare 50 μl and 100 μl aliquots in screw-cap microcentrifuge tubes and store at minus 20°C for up to 6 months. Avoid more than three freeze thaw cycles.

### 10 mM EdU solution

Add 4 ml of DMSO to EdU substrate, mix well and aliquot into 50 ul batches and store at -20C.

### Saponin based wash buffer

Add 1 ml of Saponin from Click iT kit to 9 mls of PBS for working buffer.

### DAPI solution

CAUTION: DAPI is a known mutagen and should be handled with care. The dye must be disposed of safely and in accordance with applicable local regulations.

0,1M Tris Base pH7,4 (from Sigma ref. T1503; MW = 121.14)

0,9% or 150 mM NaCl (from Sigma ref. S3014; MW = 58.44)

1mM CaCl2 (from Sigma ref. C2661; MW = 110.986)

0.5 mM MgCl2 (from Merk ref. 1.05833.1000; MW = 203.30) - 6H2O

0.2% BSA (from Sigma ref. A4503)

0.1% Nonidet P40 (from Sigma ref. I8896) - Igepal

10 μg/mL DAPI (from Invitrogen ref. D21490)

### Propidium Iodide (P4170, Sigma-Aldrich) solution

(0.5 mg/ml in PBS with 0.1% sodium azide, pH approximately 7.4)

## BOX 1

### HES media (for culture of hESc and hIPSC on ir MEF or irHFF feeder layers)

-Knockout DMEM (Gibco #10829-018) (4°C).

-Knockout Serum Replacement (KSR) (Gibco #10828-028) (-20°C).

- Non-Essetial Amino Acids 100× (Gibco) (4°C).

-2-Mercaptoethanol 50 mM (Gibco #31350-010) (4°C).

-Penicillin (10.000U/ml)/Streptomycin (10.000 μg/ml) (100X).

(Gibco #15140-122) (-20°C).

-GlutaMAX 200 mM (100X) (Gibco #35050-038) (-20°C).

- basic Fibroblastic Growth factor 100 μg/ml (Peprotech) (-20°C).

**250 ml**

-Knock-Out DMEM   193,5 ml

-20% Knock-Out serum replacement   50 ml

-1× Non-Essetial Amino Acids   2,5 ml

-50 U/ml-50 μg/ml Penicillin/Streptomycin (P/S)   1,25 ml

-1× GlutaMAX   2,5 ml

-50 μM 2-Mercaptoethanol   250 μl

-8 ng/ml basic Fibroblastic Growth factor   20 μl

### HUES media (for culture of hESC or hiPSC independent of feeder layers on matrigel)

-Knockout DMEM (Gibco #10829-018) (4°C).

-Knockout Serum Replacement (KSR) (Gibco #10828-028) (-20°C).

- Non-Essetial Amino Acids 100× (Gibco) (4°C).

-2-Mercaptoethanol 50 mM (Gibco #31350-010) (4°C).

-Penicillin (10.000U/ml)/Streptomycin (10.000 ug/ml)(100X)

(Gibco #15140-122) (-20°C).

-GlutaMAX 200 mM (100X) (Gibco #35050-038) (-20°C).

- basic Fibroblastic Growth factor 100 μg/ml (Peprotech) (-20°C).

-Human albumin 20% (Grifols) (RT).

**300 ml**

- Knockout DMEM   250 ml

-10% Knockout Serum Replacement   30 ml

-1× Non-Essetial Amino Acids   3 ml

-50 μM 2-Mercaptoethanol   0,3 ml

-1× penicillin/Streptomycin (100 U/ml, 100 μg/ml)   3 ml

-1× GlutaMAX 200 mM   3 ml

-10 ng/ml basic Fibroblastic Growth factor   30 μl

-Human albumin 20%   7.5 ml

The use of conditioned media for hESC or hiPSC on matrigel is essential. To make conditioned media, simply add HEUS media to irMEFS over night and the next day fliter through a millipore 0.45 uM filter onto cells.

## BOX 2

### Plating irHFF or irMEF cells

The day before plating ES[4] cells seed irradiated feeders HFF (ATCC) on gelatinized plates. For a 100 mm plate:

**1**. Gelatinize by adding 5 ml/dish 0.1% gelatin in water (Chemicon). Incubate at least 30 min at 37°C (you can also leave the plate with gelatin overnight at 37°C). Aspirate the gelatin solution and let the plate dry under the hood. **2**. Plate 4 × 10^6 ^cells/100 mm plate in DMEM +10%FBS, 1 × Glutamax, 1 × Pen/Strep.

### Plating hESC or hiPSC by trypsinization on matrigel coated plates

When the colonies get big enough and start to touch other colonies is time to passage them. Coating of plates using 1:15 diluted Growth Factor Reduced (GFR) matrigel (from BD) in Knockout DMEM is done the day before at 4°C and has been described previously [19].

• Aspirate media and wash cells with PBS.

• Add 1 ml of 0,05% trypsine-EDTA and incubate at 37°C for 2-5 minutes.

• Add 9 ml of HUES media and resuspend the cells:

• If you want a single cell suspension: perform a complete trypsinization and pipette up and down several times before plating.

• Plate the cells at the desired dilution, usually 1:6 dilution.

• Don't change the media the following 48 hours after plating.

• Change the media daily. Warm up just the needed media each time.

## PROCEDURE

### Maintaining ESC and iPSC in culture using matrigel or feeder layers

For hESC or iPSC on feeder layers, pluripotent cells can be trypsinized and then separated during analysis based on Forward Scatter (FSC). To maintain the line, cells must be passaged using a scrapper via a video screen linked to a stereomicroscope. Pluripotent cell colonies are scrapped off the feeder layer, collected and split into waiting feeder layers no more than 1:3 (for ES cells) and 1:10 (for iPS cells). For hESC or hiPSC maintained on matrigel trypsinization is used (**see box 2**).

CRITICAL STEP: Proper cultivation of pluripotent stem cells to keep cells healthy is essential because it can affect growth rates dramatically, which can be seen by some cell cycle analyses.

### Assessment of cell cycle profile and apoptosis by Flow Cytometry from the same sample

TIMING 3 hours.

**Figure 1:**
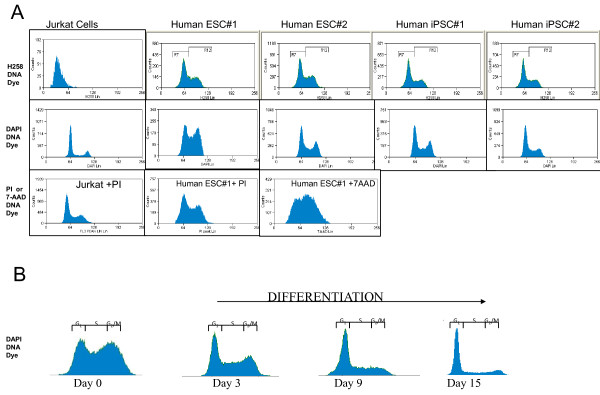
**Analysis of different DNA dyes in pluripotent cells**. Panel A: Comparison of FACs histograms of four different DNA dyes, staining of Jurkat cells, Human ESC and human IPS cells. We found that DAPI gave the best overall profile of the G1, S and G2/M phases of the cell cycle across different cell types described in the figure. Panel B: FACs histograms of DAPI staining of mouse ESC at day zero (undifferentiated pluripotent cells) and at different stages of differentiation using general differentiation cell culture conditions (Gelatin coated plates with 20% FCS in DMEM). Note the dramatic changes in the overall profile of the G1, S and G2/M phases of the cell cycle with differentiation.

We use the Invitrogen Click IT EdU kit for detection of S phase with a modified protocol to give quicker and easier to achieve results. We have tested many DNA dyes and DAPI has been found best DNA staining for pluripotent cells, especially when samples are left overnight at 4°C (Figure 1). For assessment of apoptosis we use the MitoProbe DiLC assay kit (M34151). Pluripotent cells can be split after EdU treatment into two tubes, one for EdU assessment of cell cycle profile and the other for assessment of apoptosis by DiLC (Mitochondrial membrane potential). In that way both measures can be made from the same sample.

## BOX 3

### Getting the most out of your cell cycle flow cytometry analysis

In addition to information about the percent of cells in each of the phases of the cell cycle from EdU/DAPI staining, extra information about the proliferative state of the cells can be gleaned from the analysis.

**Figure 2:**
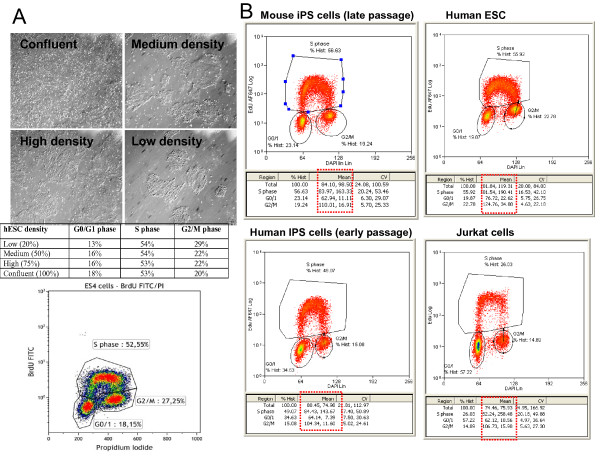
**Analysis of cell cycle**. Panel A: Photographs of hESC at different densities. The table below is an analysis of cell cycle profile using EdU for 30 minutes at 10 uM. Note the differences in G1 and G2/M with different cell density. Using cells at 50-60% confluence is optimal. *Lower Panel: *Histogram of hESC treated with BRDU demonstrating a 50-60% of cells in S phase. Panel B: Flow cytometry histograms of cell cycle profile of hESC, human IPS cells, mouse IPS cells and Jurkat cells treated with EdU and DAPI. *Note *the red box highlights the mean intensity of EdU signal that can also be used as an indicator of the proliferative state of the cells.

**(A)**. To the right of the percentages of each phase in Flow Cytometry analysis histograms there are two values (Figure 2). The first is the mean intensity of signal from DAPI and the second is the intensity of signal for EdU. The value for EdU is an indication of how fast the cells are proliferating because the faster they proliferate the more EdU they will absorb. Therefore this number can act as a guide to the S phase percentages when assessing how fast they are proliferating. Be careful to ensure to use the same concentration of EdU (10 uM) for the same time period (45 minutes exposure to cells) with the same density of pluripotent cells for all experiments. **(B)**. A second piece of information that can be taken from the cell cycle Flow Cytometry analysis is a clear separation of the 3 phases; G1, S and G2/M phases (Figure 2). A clear separation is desirable with the S phase high above the other phases. If this is not the case it means that the EdU staining did not work or the pluripotent cells are not healthy and not proliferating normally (they could be quiescent. Ensure to check that the pluripotent cells are healthy before experiments.

### Assessment of cell cycle profile by flow cytometry

CRITICAL STEP Make sure pluripotent cells are between 40-60% confluent in all experiments because cell density affects the final results (Figure 2).

1. Label the cells with EdU substrate by adding 10 ul of 10 mM EdU solution to 10 mls of culture medium in 10 cm plate of cells (a 1:1000 dilution) to make a 10 uM final working concentration and incubate for 45 minutes at 37°C 5% CO2. See Table 1.

**Table 1:** Modified working volumes for Click IT cocktail for EdU analysis of S phase.

Reaction components for Click IT cocktail		Number of reactions	
	**2**	**5**	**10**

10× Click-IT Reaction Buffer	87.5 ul	219 ul	438 ul

CuSO4	20 ul	50 ul	100 ul

Fluorescent dye	5 ul	12.5 ul	25 ul

10× Reaction buffer additive	10 ul	25 ul	50 ul

Water	877.5 ul	2193.5 ul	4387 ul

TOTAL volume	1 ml	2.5 ml	5 ml

2. We have found that for pluripotent cells leaving the EdU for 45 minutes is optimal[4].

3. Wash cells once with PBS and harvest cells by trypsinization for pluripotent cells cultured on matrigel or by scrapping the cells off the feeder layer to get pure pluripotent cell population. Timing 10 minutes.

4. Wash the cells again in PBS and transfer cells to Flow Cytometry tubes. Spin down at 1500 rpm for 2 minutes to form pellet. Remove PBS simply by turning tube upside down into a waste container with one quick movement, drying the edges on paper to remove drops at edge of tube. This leaves approximately 50 ul of PBS in the FACS tube.

CRITICAL STEP: Tapping the tube only once upside is essential otherwise the cells will slide out.

CAUTION: EdU cocktail breaks down GFP signal that may be expressed in genetically modified pluripotent cells being used, but not the Alexa fluor dyes. Therefore consider adding an anti-GFP AlexaFluor-488 conjugated antibody step to cells before labelling with EdU cocktail to retrieve the GFP signal.

5. Add 100 μl of Click IT fixative for 15 minutes R/T and mix well by vortex.

6. Wash once 3 mls of PBS and pellet cells again at 1500 rpm for 2 minutes and discard wash buffer by inverting the Flow Cytometry tube as described above.

7. Permeabilize the cells by adding 100 μl of saponin based wash buffer for 30 minutes R/T.

8. Wash with 3 mls of saponin based wash buffer and pellet cells again.

9. Add **Click IT reaction cocktail **and add 500 μl per Flow Cytometry tube and incubate for 30 minutes R/T in the dark.

10. Wash once with PBS, pellet cells and add **DAPI solution **and incubate overnight at 4°C and analyse the following day for best results or leave at 4°C for at least 2 h for immediate FACS analysis.

CRITICAL STEP: We have tried different DNA dyes (PI, Hoechst33342, Click-iT EdU Cell Cycle 633-red, 7AAD, and in our hands the best cell cycle profiles in terms of lowest coefficients of variation for the G0/1 peak came up when using DAPI.

11. Assess a minimum of 10000 events at levels described in Table 2.

**Table 2:** MoFlo Instrument Settings the Flow Cytometry machine for detection of GFP, EdU and DAPI signals.

Laser	Excitation wavelength & power	Band Pass Filters used for collection of emitted fluorescence (wavelength/power)
**Blue**	488 nm at 30 mWatts	Green emission - 530/30 (from anti-GFP/Alexa Fluor 488)

**Ultra violet**	351 nm at 20 mWatts	Blue emission - 450/65 (from DAPI)

**Red**	633 nm at 35 mWatts	Red emission - 670/20 (from Alexa Fluor 647 azide linked to EdU)

CRITICAL STEP: Check to ensure that you flow cytometry machine has PMT detectors that are Hamamatsu -15, which are very sensitive. If not higher powers of 100-200 mW may be needed to detect DAPI.

### Assessment of Apoptosis using DiLC by Flow Cytometry analysis

**Figure 3:**
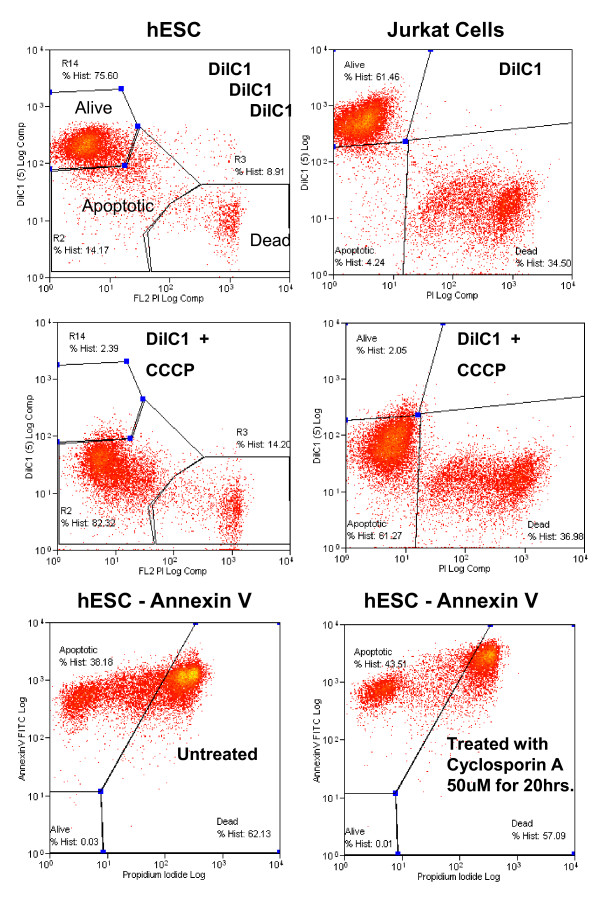
**Analysis of Apoptosis using DiLC and PI**. Comparison of two different methods for measurement of apoptosis, DilC and Annexin V in human ESC (pluripotent cells) or Jurkat cells (Differentiated cells). Flow cytometry histogram of hESC assessed for apoptosis levels using DiLC, which measures the mitochondrial membrane potential. Note that the control ESC treated with CCCP (with DiLC at the same time) to show the shift in signal, demonstrating that the assay is working. Cyclosporin A was used in Annexin V assays to induce apoptosis (50 uM for 20 hours).

We have assessed two methods for measuring apoptosis in pluripotent cells including DiLC and Annexin V. We found that DiLC gave the best results. Annexin V is not suitable for trypisination of pluripotent cells because it causes false positives (see Figure 3 and Table 3).

**Table 3:** MoFlo Instrument Settings for detection of DilC and PI signal.

Laser	Excitation wavelength & power	Band Pass Filters used for collection of emitted fluorescence (wavelength/power)
Blue (PI)	488 nm at 30 mWatts	Orange emission - 580/30 (from PI)
		Red emission - 670/30 (from PI)

Red (DilC)	633 nm at 35 mWatts	Red emission - 670/20 (from DilC)

TIMING 2 hours.

12. The other sample of pluripotent cells split from the original is used to assess levels of apoptosis.

13. Use the MitoProbe DiLC Assay Kit for Flow Cytometry (M34151) from Invitrogen to measure mitochondrial membrane potential.

14. Tripsinize or scrape pluripotent cells from plate and pellet cells by centrifuge and place in pre-warmed (37°C) PBS.

CRITICAL STEP Maintain the cells at 37°C at all times in a water bath for stable membrane potential. Having access to a water bath in the flow cytometry machine room is advised.

15. Wash cells again in 1 ml pre-warmed PBS in Flow Cytometry tubes and pellet.

16. For control tubes add 1 uL of 50 mM CCCP and incubate cells at 37°C for 5 minutes.

CRITICAL STEP: Including a CCCP (it is a metabolic inhibitor) control is essential to clearly define within the histogram analysis the cut-off between the apoptotic and alive populations in the first experiments and to determine that the apoptosis assay is working. Adding CCCP causes loss of mitochondrial membrane potential (MMP) and should cause a shift of cells out of the top left where alive cells can be found (Figure 3).

17. Add 5 uL of 10 μM DilC (can be added at same time with CCCP for controls) and incubate at 37°C, 5% CO_2 _for 30 minutes).

18. Wash with 3 mls of pre-warmed PBS and pellet cells and discard wash by tipping once and drying edges (50 uL of solution is left with this method).

19. Resuspend cells with 100 uL of propidium iodide (PI) solution and incubate for 15 minutes at 37°C.

20. Proceed to analyser to assess percent of cells apoptotic/alive/dead (see Figure 3).

21. Assess a minimum of 10,000 events.

CRITICAL STEP: samples must be analysed immediately to ensure the membrane is preserved. Heating the DAKO MoFlo flow cytometer tubing to 37°C is recommended. With the MoFlo, the sample port can be set at any temperature with a range 0°C- 40°. Because the 37°C is important in order to maintain the MMP, taking advantage of this feature in your flow cytometry machine will help maintain valid results.

### Western Blot methodology

**Figure 4:**
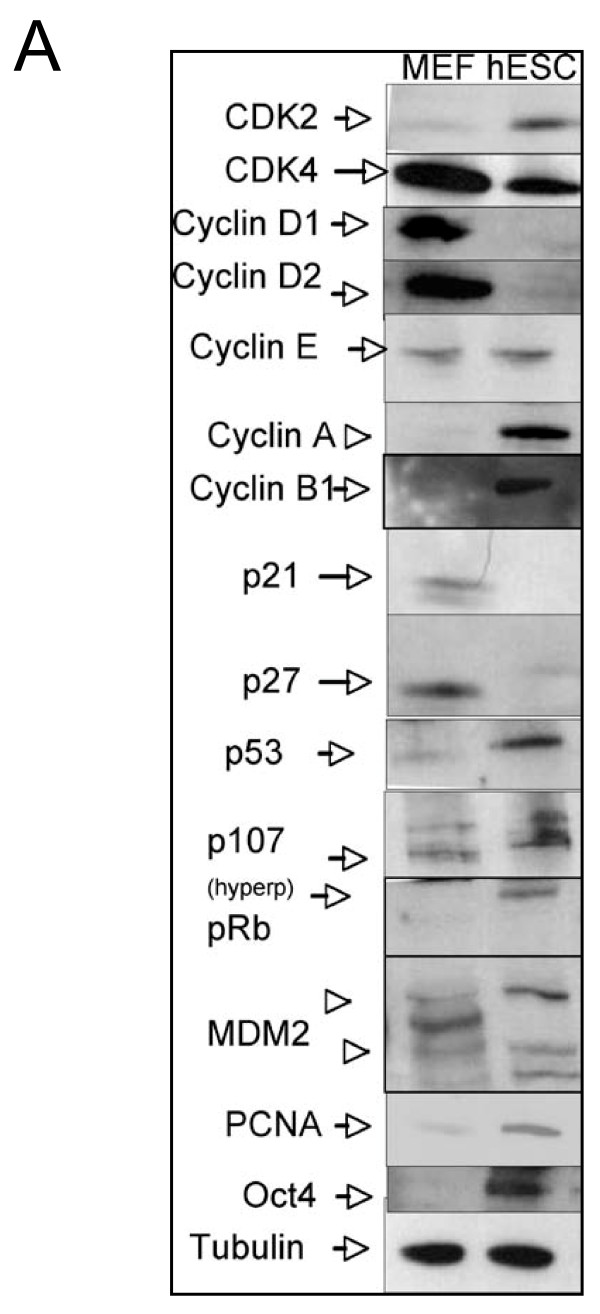
**Western blot analysis of cell cycle proteins**. Western blot of fourteen cell cycle antibodies described in table 4 using irradiated MEFs as a control and undifferentiated human ESC protein. Alpha-tubulin used as a loading control and Oct4 is used to determine pluripotent state. Note: a comprehensive analysis such as this one can be achieved in one week. sc: Santa Cruz; Cell Sign: Cell Signal antibodies.

We include a list of antibodies that have been tried and tested for detecting cell cycle proteins and apoptosis proteins in pluripotent cells (Table 4). These selected antibodies, of some of the main cell cycle proteins, work with resolving SDS gels of 4-12% using between10-15 μg of total protein unless specified in table 4. If non resolving gels are used then use 8% gels for Rb, p107, cdc27 and MDM2, which are 80 Kda or bigger proteins and 12% SDS gels for all others. See Figure 4 for an example of the expected results for pluripotent cells using some of these antibodies. Using 10 well gels, up to four lysates can be loaded twice on one gel, with protein molecular weight markers and four gels run at the same time (two gels per container). Each membrane can be blotted twice for two different antibodies from different species (i.e. first with mouse antibodies, and developed and then re-blotted with the rabbit primary antibodies and developed again) and a final time for alpha tubulin as a loading control (not listed). Therefore, up to 16 antibodies can be tested from the 4 gels using between 40 and 60 μg of total protein. If manipulating a cell cycle gene by gain of function (cDNA) or loss of function (shRNA), then protein lysates can be run alongside the ESC lane to determine the overall effect on core cell cycle protein expression levels.

**Table 4:** Tested and working antibodies for analysis of cell cycle and apoptosis by western blot methodology.

Antibody name	Company and Cat #	Dilution and expected size (KDa).
P53	sc-126(DO-1) mouse	1:1000 5%milk/TBS. 53 KDa
P19ARF	R562 Abcam Rabbit	1:1000 5%milk/TBS 25 Kda (30-50 μg)
P16INK4a	M-156 (sc-Rabbit)	1:1000 5%milk/TBS 16 Kda
Rb	C15 (sc rabbit)	1:1000 5%milk/TBS 105 KDA
P107	C18 (sc Rabbit)	1:1000 5%milk/TBS. 107 KDa
Cyclin A	C19 (sc-rabbit)	1:1000 5%milk/TBS 54 Kda
Cyclin A1	H-230 (sc rabbit)	1:1000 5%milk/TBS 54 Kda
Cyclin A2	Epitomics E399 (rabbit)	1:500 5%milk/TBS 54 Kda
Cyclin B1	Sc-245 (mouse)	1:1000 5%milk/TBS 60 Kda
Cyclin D1	H-295 (sc rabbit)	1:1000 5%milk/TBS 34 KDa
Cyclin D2	M20 (sc rabbit)	1:1000 5%milk/TBS 34 KDa
Cyclin E	M20 (sc rabbit)	1:1000 5%milk/TBS 53 KDa
E2F1	Sc-251 (sc mouse)	1:1000 5%milk/TBS 55 KDa
P21CIP	F5 (sc mouse)	1:1000 5%milk/TBS 21 KDa
P27KIP	(M-197) sc776 rabbit	1:1000 5%milk/TBS 27 KDa
CDK4	C22 (sc rabbit)	1:1000 5%milk/TBS 34 KDa
CDK2	H-298 (sc rabbit)	1:1000 5%milk/TBS 34 KDa
PCNA	PC-10 (sc. Mouse)	1:1000 5%milk/TBS 36 KDa
MDM2	Abcam 2A10 (mouse)	1:1000 5%milk/TBS 80 KDa(30-50 μg)
Cleaved Casapse 3	Cell sign (rabbit) #9664	1:1000 5%milk/TBS 18 KDa
Cdc27	C4 (sc mouse)	1:1000 5%milk/TBS 102 KDa

TIMING: 1 week.

## Discussion

Accurate cell cycle analysis has to be based on data from cells handled in a manner in which the least variation between experiments occurs. In our hands the reliability and validity of the described method for measurement of apoptosis and cell proliferation in the same sample is very good. In the past other methods have been developed to measure cell proliferation that provide an alternative approach (see trouble shooting table 5). Specifically, the use of other DNA dyes may improve the method in different laboratories with different machines. Other DNA specific dye, such as propidium iodide (PI) can be added to the labelled cells for two-parameter analysis of EdU. We settled on DAPI (overnight at 4°C) giving the most reliable results. In our experience with EdU stainings for flow cytometry analysis, the CVs have remained low and reproducible with DAPI staining and not 7-AAD (a modification from the kit). To achieve this we recommend that factors such as cell culture conditions are maintained well, exposure time of substrate and concentration (as well as storage of substrate) is maintained well and that cell density is equal between experiments (see Figure 1). Other methods for assessment of cell cycle have been published before. Krishan and Hamelik have modified the EdU procedure as an alternative method to the one we have described (20). In comparison to our method, the method involves a more laborious step of isolation of cell nuclei. Moreover, the requirements and complexicity of the method is more demanding. Therefore, to maintain simplicity and ease in use of the protocol with low CVs we prefer to use DAPI staining with EdU, and give the same results compared to using BRDU (see Figure 2).

**Table 5:** Troubleshooting

Step	Problem	Possible reason and solution
	Low number of cells in S Phase	EdU substrate incubation too short or batch of substrate used too many times. Use fresh aliquoted EdU and incubate 15-30 minutes longer.
	No distinct G1/S/G2M phases	Cells not healthy or DAPI staining not working. Check that cells look healthy before starting experiment and leave DAPI staining overnight at 4°C (in the fridge). Also consider using a modified method using Hamelik and Krishan protocol by lysing cells (Hypotonic solution) for 1 minute followed by centrifugation and then using EdU reaction (Click iT)[20].
	DilC % apoptosis lower than expected or not working.	Check with CCCP control there is a shift in DilC positive cells to negative. Keep cells at 37°C at all times throughout the procedure and analyse immediately following PI staining.

We chose EdU click IT kit over standard BRDU staining for S phase analysis because it gave reliable results and was quicker to complete with the same results (Figure 2). It is important to note that the Click-it method breaks down GFP signal so we recommend to use an anti-GFP antibody to recognize GFP expression, since it uses copper sulfate and ascorbate to develop a signal. Other methods have suggested to fix the cells in dilute paraformaldehye which keeps the GFP intact prior to staining (21). We agree that fixing cells with dilute paraformaldehyde can preserve the GFP signal, however in our hands, using an anti-GFP AlexaFluor-488 antibody, a flurochrome that is not degraded during the reaction cocktail step, helps to retrieve the GFP signal, a significant advantage with pluripotent cells that are so often hard to infect/transfect. Given that ESC are notoriously difficult for gain and loss of gene function studies, retrieving the GFP signal with an Alexaflour 488 GFP antibody, gives an advantage to attaining the best results form flow cytometry work.

Flow Cytometry machines available to researchers from lab to lab will vary and that different machines will require different power settings. The machine used in this protocol is a High Performance MoFlo Cell sorter. The MoFlo is equipped with 3 lasers, an Innova 90C UV laser from Coherent, a red diode 635 nm and Lytcyt200s 488 nm solid-state laser. The PMT detectors used are Hamamatsu -15; this is the most sensitive model and therefore low power levels at 30 mW are used. If we work with 100-200 mW of power the signal would be out of range. The best resolution of DNA signals may require more power using different machines; however, this would depend on DNA dye used. For example Hoechst dye exclusion assays to identify the side population in a bone marrow just require 20-30 mW of UV power using a machine not equipped with a Hamamatsu-15 detector.

The faster cells proliferate when the cells are exposed to BrdU or EdU for a fixed amount of time means more cells labeled in late S, G_2 _or M phases. The main data from EdU staining is S phase percentages but we thought to also highlight that the mean intensity of signal can also be used a secondary guide as well. When working with a fixed amount of time and EdU concentration as recommended in our protocol, the difference in the EdU Signal to Noise ratio (S/N) can be directly related with how fast they are growing. The more time exposure and the more concentration of EdU the higher the S/N ratio and for this reason it is important to use pluripotent cells at around 50-60% confluency, the same concentration of EdU and also the same incubation time for all the treated samples. We recommend that 10 uM EdU and 45 minutes to be used across the field for pluripotent cells. In that way coefficient of variation can be calculated intra-laboratory for many different pluripotent cell lines and we have found very little variation between experiments when this protocol is followed correctly.

## Conclusions

At the end of the protocol a comprehensive analysis of the percent of cells in G_0_/G_1 _phase, S phase and G_2_/M phase should reveal an expected percentages of cells in each phase as: 15%/65%/20% +/- 5%, respectively. Changes greater than 5% in the G_1 _or G_2_/M phases between samples of interest are considered significant. Further evidence of an effect on cell cycle can be done by performing a proliferation growth curve to show differences more clearly. In our experience, differences even less than 10% in S phase can be seen in proliferation curves maintained for over 2 to 3 weeks. Western Blot analysis should reveal a protein expression for the main cell cycle proteins as seen in Figure 4. Apoptosis measurements by FACs should give a normal range of 70-75% of cells alive, 20% in transition and 5-10% apoptosis using DilC combined with PI methods. Overall, this protocol will give rise to a detailed analysis of the cell cycle and apoptosis status in human and mouse pluripotent cells, offering a standardized approach across the field. For more details of making lentivirus and infection of ESC, please see previous published work from our group (17-19).

## Competing interests

The authors declare that they have no competing interests.

## Authors' contributions

MJE designed the overall protocol, wrote the manuscript after obtaining material from all authors. JMAV developed the flow cytometry protocol and performed low cytometry experiments. CM developed and tested the western blot methods. JCIB supervised the project.

All authors have read and approved the final manuscript.
